# Simulation study on the thermal effect of continuous laser heating quartz materials

**DOI:** 10.3389/fchem.2024.1435562

**Published:** 2024-07-23

**Authors:** Wei Li, Jichuan Wu, Yanglong Li, Lingyuan Wu, Bo Fu

**Affiliations:** Institute of Fluid Physics, China Academy of Engineering Physics, Mianyang, China

**Keywords:** CW laser heating, quartz materials, temperature field distribution, thermal effect, finite element simulation

## Abstract

The continuous development and application of laser technology, and the increasing energy and power of laser output have promoted the development of various types of laser optical systems. The optical components based on quartz materials are key components of high-power laser systems, and their quality directly affects the load capacity of the system. Due to the photothermal effect when the laser interacts with the quartz material and generates extremely high temperatures in a short period of time, it is impossible to experimentally solve the phenomena and physical mechanisms under extreme conditions. Therefore, it is very important to select a suitable method to investigate the thermal effect of intense laser interaction with quartz materials and explain the related physical mechanism. In this study, a three-dimensional quarter-symmetric laser heating quartz material geometry model by using nonlinear transient finite element method was established, and its transient temperature field distribution of the quartz material after being heated by a 1,064 nm continuous laser was investigated. In addition, the influence of different laser parameters (laser spot radius, heat flux and irradiation time), material parameters (material thickness, material absorption rate of laser) on the thermal effect of heating quartz material were also studied. When the laser heat flux is 20 W/cm^2^, the diameter of the laser spot is 10 cm, the irradiation time is 600 s and the thickness is 4 cm, the temperature after laser heating can reach 940.18°C, which is far lower than the melting point. In addition, the temperature maximum probes were set at the overall model, spot edge and rear surface respectively, and their temperature rise curves with time were obtained. It is also found that there is a significant hysteresis period for the rear surface temperature change of the quartz material compared with the overall temperature change due to heat conduction. Finally, the method proposed can also be applied to the laser heating of other non-transparent materials.

## 1 Introduction

Laser has the advantages of stronger coherence, directivity, monochromism and high brightness, and plays an important role in many fields such as industry, agriculture, medical treatment, national defense, military and scientific research (Boneberg and Leiderer, 2022; Zhao et al., 2022; Li X. et al., 2023; Liang S. R. et al., 2023; Lai et al., 2023; Li et al., 2024a). In the industry, laser is mainly used for material processing, cleaning and cutting (Armbruster et al., 2023; Liang C. et al., 2023; Dang et al., 2023; Alkunte et al., 2024). In the national defense and military, lasers are widely used in the field of laser weapons (Li et al., 2024b; Soni et al., 2024). Since then, the energy and power of laser output have been continuously improved with the continuous development and application of laser technology, which has promoted the development of various laser optical systems, such as high-power laser systems and laser processing systems (Khalatpour et al., 2021; Fraggelakis et al., 2022; Wolf et al., 2022). The optical element based on quartz material is the key component of high-power laser system, and its quality directly affects the load capacity of the system. The excellent load capacity ensures the safe and stable operation of the system under the action of high-power laser for a long time. If some areas of the optical elements absorb too much laser energy, thermally induced damage may occur, even endangering the safe operation of the high-energy laser system, thus limiting the development of the system towards high power and energy density (Zhu et al., 2018; Fourmaux and Kieffer, 2021; Luo et al., 2024). Therefore, the study of the thermal effects of laser interaction with quartz materials and the enhancement of quartz materials’ resistance to laser irradiation are of great application value.

Due to the photothermal effect when the laser interacts with the quartz material and generates extremely high temperatures in a short period of time, it is impossible to experimentally solve the phenomena and physical mechanisms under extreme conditions (Vidal et al., 2020; Merkininkaitė et al., 2022; Liang et al., 2024). Therefore, it is very important to select a suitable method to study the thermal effect of intense laser interaction with quartz materials and explain the related physical mechanism. The current common research methods include theoretical analysis, numerical simulation and experimental method (Sihn et al., 2016; Chen et al., 2017; Elsheikh et al., 2021). Chen et al., (2017) carried out a 2D modeling work on the process of repeated long-pulse laser heating materials, and used the variable separation method combined with the Laplace transform to obtain the analytical solution of the 2D modeling of temperature distribution, and found that the results of the analytical solution were consistent with the existing finite element method, providing a certain theoretical basis for revealing the mechanism of laser heating. Sihn et al., (2016) investigated the complex heat transfer process and damage evolution of 1,070 nm continuous laser irradiation of the nickel-based alloy Inconel 718 by means of a finite element numerical simulation method combined with the corresponding experimental work, predicted the onset and progression of the material loss process and verified the prediction experimentally. Elsheikh et al., (2021) investigated the physical process of moving continuous laser heating of a finite steel plate, and obtained the laser heating process temperature distribution by solving the Green’s function, and the results coincided with the experimental results under the same laser conditions. Lei et al., (2024) investigated the process of large-area and continuous heating of ceramic materials by a flat-top continuous laser based on the finite element method, established a 3D simulation model of the heating progress. The effects of different laser and geometrical parameters on the heating process were investigated. Their methods can also be applied to the laser heating process of non-transparent materials. Wang et al., (2020) investigated the fused silica polishing process as well as the physical mechanism of the Gaussian and flat-top laser beams and further optimized the laser polishing process through experiments and numerical simulations, respectively. The experimental and simulation results show that lower laser power density and slow laser scanning speed can improve the processing quality of the laser polishing process. It can be found from the above research work that the theoretical analytical method can accurately calculate the temporal and spatial distributions of the temperature field after intense laser irradiation of an optical material, although its model is too simple and difficult to match the experimental results. The experimental method is costly and has strict environmental requirements, which makes it difficult to explain the experimental phenomena under extreme conditions. The numerical simulation method can not only overcome the shortcomings of the first two, but also its simulation results can guide the experimental process, reduce the experimental steps and save resources, which is the main method to study the interaction between the strong laser and the quartz material at present (Tan et al., 2021; Lei et al., 2024).

In this study, a 3D quarter-symmetric geometrical model by using nonlinear transient finite element method was developed, and its transient temperature field distribution of the quartz material heated by a 1,064 nm continuous laser using a surface laser heat source was investigated. In addition, the influence of different laser parameters (laser spot radius, heat flux and irradiation time), material parameters (material thickness, material absorption rate of laser) on the thermal effect of laser heating quartz materials were also studied. The temperature maximum probes were set at the overall model, spot edge and rear surface respectively, and their temperature rise curves with time were obtained. It is also found that there is a significant hysteresis period for the rear surface temperature change of the quartz material compared with the overall temperature change due to heat conduction. Finally, the loss of heat through thermal convection and thermal radiation of the heating process were also took into account, making the results more reliable. This work not only provides theoretical support for the study of the thermal effects of quartz materials under continuous heating conditions of high-energy lasers, but also guides the experimental process based on the results of the simulation study. However, this work does not consider the substantial changes and phase transitions on the surface of quartz materials during the heating process, which may lead to relative errors between the simulation results and the experimental results, but does not affect the correctness of the results.

## 2 Geometric model and simulation method

### 2.1 Geometric model

**FIGURE 1:**
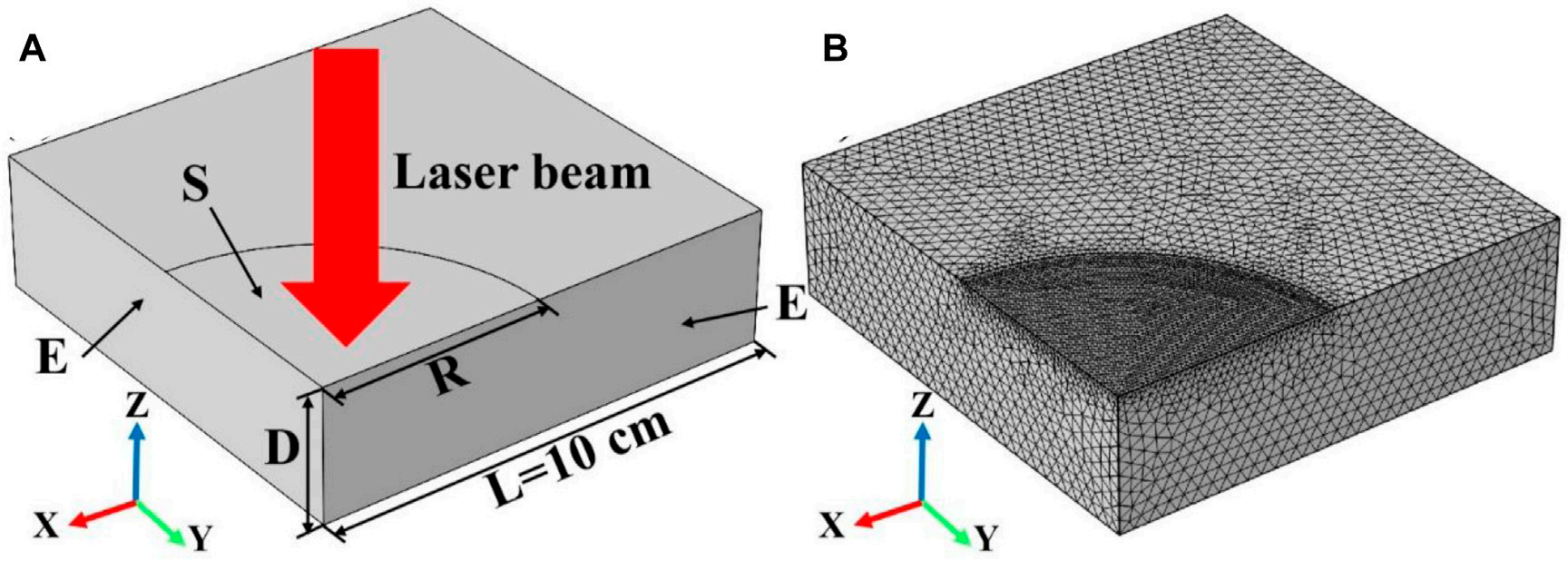
**(A)** shows the schematic diagram of the laser heating simulation model, where the symbols “D”, “E”, “R”, and “S” indicate the thickness of the model, the symmetric boundary condition, the radius of the laser beam, and the surface heat source, respectively; **(B)** shows the mesh division of the laser heating simulation model.

The schematic diagram of the simulation model of laser-heated quartz material is illustrated in Figure 1A. A 3D quarter-symmetric model was used to simulate the change of the temperature field of quartz material caused by continuous laser irradiation. The geometric thickness, length and width of the quartz material are 4 cm, 20 cm and 20 cm, respectively. The laser parameters used in the simulation are as follows: the laser wavelength is 1,064 nm, the laser heat flux is 20 W/cm^2^, the laser radius is 5 cm, the laser irradiation time is 600 s, and the laser absorptivity is numerically the same as the surface emissivity of the quartz material. Besides, the radius direction is set as a symmetric boundary condition, and the model can be simulated according to the whole quartz material during the calculation process. The symbol “D,” “E,” “R,” and “S” indicate the thickness of the model, the symmetric boundary condition, the radius of the laser beam and the surface heat source respectively. Finally, the mesh division of the simulation model is shown in Figure 1B. In order to improve the accuracy of the simulation results, the meshing of the laser spot region is relatively dense, and the meshing of the other regions of the model is sparse.

### 2.2 Simulation method

The laser heating of quartz materials involves some complex physical phenomena, so it’s necessary to make several assumptions about the heating process to make the model simpler and more reliable. The corresponding assumptions are as follows (Shukla and Lawrence, 2014; Lei et al., 2024; Luo, 2024):

• The laser source is the surface heat source;
• Material oxidation reactions are not involved in the heating process;
• The quartz material is isotropic;
• Boundary conditions: thermal convection losses and thermal radiation losses are considered on the laser-heated surface, and the rest of the surface is adiabatic.

In addition, the model proposed is applicable to the case of laser heating without phase transformation of the material and without substantial changes in the surface. A three-dimensional quarter-symmetric geometrical model of a laser-heated quartz material by using the nonlinear transient finite element method was established, and its distribution of the transient temperature field of a quartz material after continuous laser heating at 1,064 nm was investigated. Under the given boundary conditions, the dependent variable is solved by solving the transient Fourier heat transfer equation with the expression, as shown in Formula 1 (Chen and Hong, 2022; Liang S. et al., 2023):

$$\rho C_{p}\frac{\partial T}{\partial t}-\nabla \left(k\nabla T\right)=Q$$ (1)

where *ρ* denotes the density of the quartz material, *C*_*p*_ denotes the heat capacity of the quartz material, and *k* denotes the thermal conductivity of the quartz material. *T* denotes the transient temperature field of the quartz material during the laser heating process, and *Q* denotes the heat source per unit volume. Due to the large heating area of the quartz material and the thickness of the specimen, the laser heating source is approximated as a surface heat source (Dittrich et al., 2022). In order to be closer to the actual heating process, we considered the thermal convection loss and thermal radiation loss of laser heating, and the corresponding boundary condition equations are as follows, as shown in Formula 2 (Lei et al., 2024):

$$n\cdot \left(k\nabla T\right)=q_{in}+h\left(T_{amb}-T\right)+\varepsilon \sigma \left(T_{amb}^{4}-T^{4}\right)$$ (2)

The normal vector is denoted by “***n***” pointing away from the surface boundary, the heat flux of the incident laser is represented by “*q*_*in*_,” and “*h*” stands for the convective heat transfer coefficient. The ambient temperature is symbolized by *T*_*amb*_ (usually assumed to be 300 K), and the surface emissivity of the quartz material is denoted by “*ε*.” Boltzmann’s constant is denoted by “*σ*,” with a value of 5.67 × 10^8^ W/(m^2^·K^4^). Apart from the surface where the laser is applied, the other surface conditions for the quartz material are adiabatic, and the corresponding boundary equation is, as shown in Formula 3:

$$n\cdot \left(k\nabla T\right)=0$$ (3)

When the laser is incident on the surface of the quartz material, a portion of the energy is absorbed and the remaining energy is reflected. The absorptivity *α*) of the material indicates how much of the laser energy is absorbed. Hence, the laser-induced heat flux (*q*_*in*_) can be described by the following equation, as shown in Formula 4:

$$q_{in}=\alpha \frac{p_{in}}{S}$$ (4)

Where *α* denotes the spectral absorbance of the quartz material at the incident laser wavelength of 1,064 nm, the symbol *P*_*in*_ represents the laser power that occurred during the incident, while S represents the area of the spot where the incident laser takes place, as shown in the following equation, as shown in Formula 5:

$$S=\frac{\pi D^{2}}{4}$$ (5)

where *D* denotes the spot diameter of the incident laser. Besides, Table 1 lists the description and abbreviations of the relevant parameters and their dimensions for the quartz material, the laser and the geometrical model.

**TABLE 1:** The description and abbreviations of the relevant parameters and their dimensions for the quartz material, the laser and the geometrical model (The thermal property parameters of quartz materials are all from the Materials Properties Database of the commercial software COMSOL Multiphysics).

Parameters	Symbol	Value	Unit
Density	ρ	2,219.39	$\text{kg}/m^{3}$
Heat capacity	$C_{p}$	62.31 + 1.93 * T + 0.004 * T^2^ − 1.70 * 10^−5^ * T^3^ + 1.88 * 10^−8^ * T^4^ − 7.06 * 10^−12^ * T^5^ (130 K < T < 925 K) 891 + 0.37 * T − 1.11 * 10^−4^ * T^2^ + 3.14 * 10^−8^ * T^3^ (925 K < T < 2,000 K)	J/(kg·K)
Thermal conductivity	k	−0.98 + 0.02 * T − 5.29 * 10^−5^ * T^2^ + 7.55 * 10^−8^ * T^3^ − 5.01 * 10^−11^ * T^4^ + 1.31 * 10^−14^ * T^5^ (273 K < T < 1,500 K)	W/(m·K)
Emissivity	ε	0.72 + 5.5 * 10^−5^ * T (273 K < T < 1,500 K)	—
Convection coefficient	*h*	5	W/(m^2^·K)
Laser wavelength	λ	1,064	nm
Laser spot radius	*r*	5	cm
Incident heat flux	$q_{in}$	20	W/cm^2^
Quartz material thickness	d	4	cm

## 3 Results and discussions

### 3.1 The effect of laser heat flux on laser heating

**FIGURE 2:**
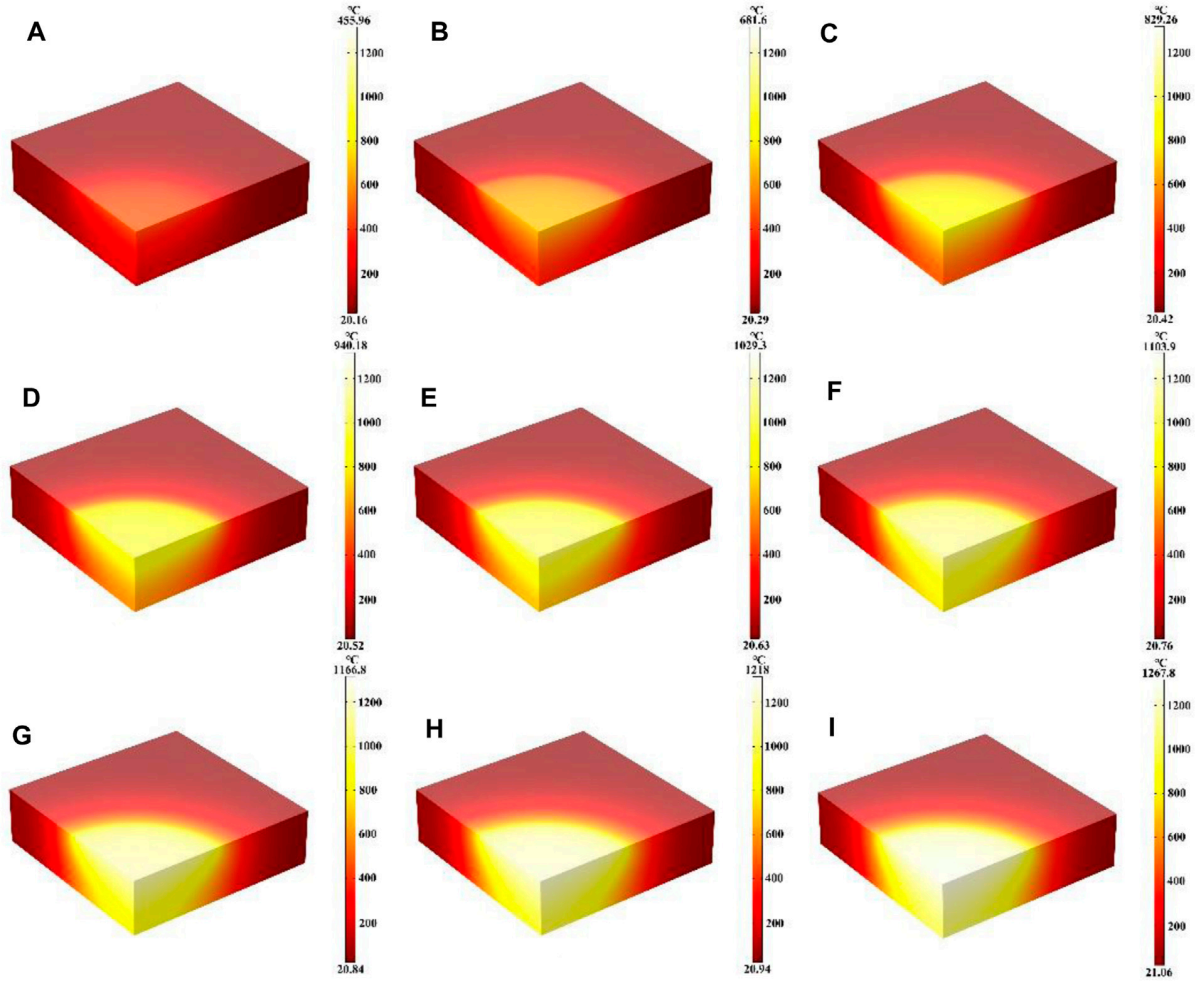
**(A–I)** Are the schematic diagrams of the model temperature distribution under different laser heat flux, respectively.

This work mainly studied the influence of different laser heat fluxes on the heating process, where the laser radius, laser absorption rate and heating time 600 s remain unchanged. Figures 2A–I shows the temperature field distribution of the quartz material after laser heating for 600 s with laser heat fluxes from 5 W/cm^2^ to 50 W/cm^2^, respectively. It can be seen that the temperature of the quartz material increases with the increase of the laser heat flux, and the temperature maximum is located at the center of the quartz material. In addition, the temperature gradient distribution inside the quartz material increases further with increasing laser heat flux. This is because the higher the incident laser heat flux, the more laser energy is absorbed by the quartz material at the same laser radius and absorption rate, which is therefore macroscopically manifested as a higher temperature of the quartz material and a larger temperature gradient (Ma et al., 2023a; Li et al., 2024c).

**FIGURE 3:**
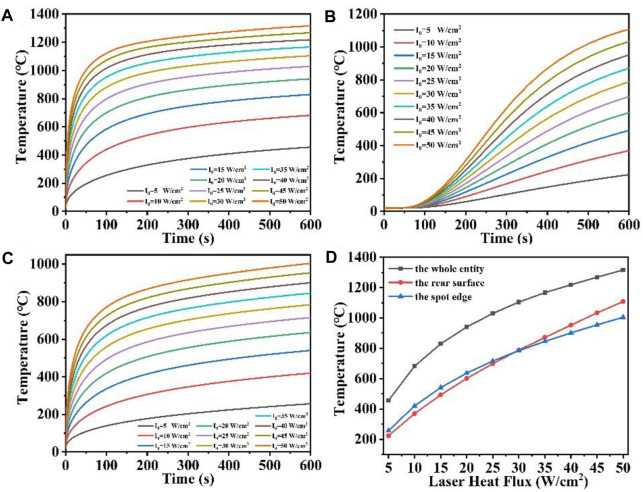
**(A–C)** Are the temperature versus time curves of the whole entity, the rear surface, and the spot edge of the model under different laser heat flux, respectively; **(D)** is the temperature versus laser heat flux curve under different positions of the model.

To more directly represent this temperature change, we set the whole quartz material temperature maximum probe, the rear surface temperature maximum probe and the spot boundary temperature maximum probe, respectively, and obtained their temperature rise curves with time. Figure 3 shows the maximum temperature versus time curves for the whole quartz material, the rear surface and the spot edge at different laser heat fluxes, respectively. The Figure 3D shows the maximum temperature versus laser heat flux curves for the quartz material at different positions. As can be seen from Figure 3A, when the laser heat flux is larger, for example, *q*_*in*_ = 50 W/cm^2^, the temperature rises rapidly by 1,200°C. As the temperature rises, the heat radiation losses begin to intensify, slowing down the temperature rise, and the temperature begins to rise slowly; when the laser power is smaller, for example, *q*_*in*_ = 5 W/cm^2^, the temperature increases at a slower rate with longer irradiation times when compared to the higher laser heat flux. Additionally, the rate of temperature rise caused by radiation loss is also reduced. Nonetheless, the higher laser power signifies a greater amount of laser energy being directed onto the surface of the quartz material with the laser spot area remaining constant, leading to increased absorption of laser energy by the quartz material (Zhang Y. C. et al., 2022; Li et al., 2024d). Under the same laser irradiation time, the greater the laser heat flux, the faster the temperature rises. Therefore, Figure 3A shows that the greater the laser heat flux, the steeper the slope of the curve of the rapidly rising temperature region. The gentler the temperature saturation curve is. From Figure 3B, it can be seen that the higher the incident laser heat flux, the higher the maximum temperature of the rear surface. In contrast to the overall maximum temperature shown in Figure 3A, there is a “hysteresis period” observed in the variation of the maximum temperature of the rear surface, and the temperature of the rear surface gradually starts to increase when the irradiation time is over 60 s, which is related to the thermal conductivity of the material (Zhang et al., 2023). In addition, Figure 3C shows that the temperature rise at the edge of the spot under different laser heat fluxes is similar to the overall temperature change in Figure 3A. Due to the stepped distribution of the flat-top laser at the edge of the spot, heat conduction is easy to occur at the edge of the spot, and the temperature at the edge of the spot is often lower than the temperature inside the laser spot under the same heat flux (Lei et al., 2024). Finally, Figure 3D shows the relationship between the temperature of the whole quartz material, the rear surface and the edge of the spot and the laser heat flux. When the laser heat flux is over 30 W/cm^2^, the maximum temperature on the rear surface is higher than the maximum temperature at the spot edge, and when the laser heat flux is less than 30 W/cm^2^, the maximum temperature on the rear surface is lower than the maximum temperature at the spot edge.

### 3.2 The effect of laser absorption on laser heating

**FIGURE 4:**
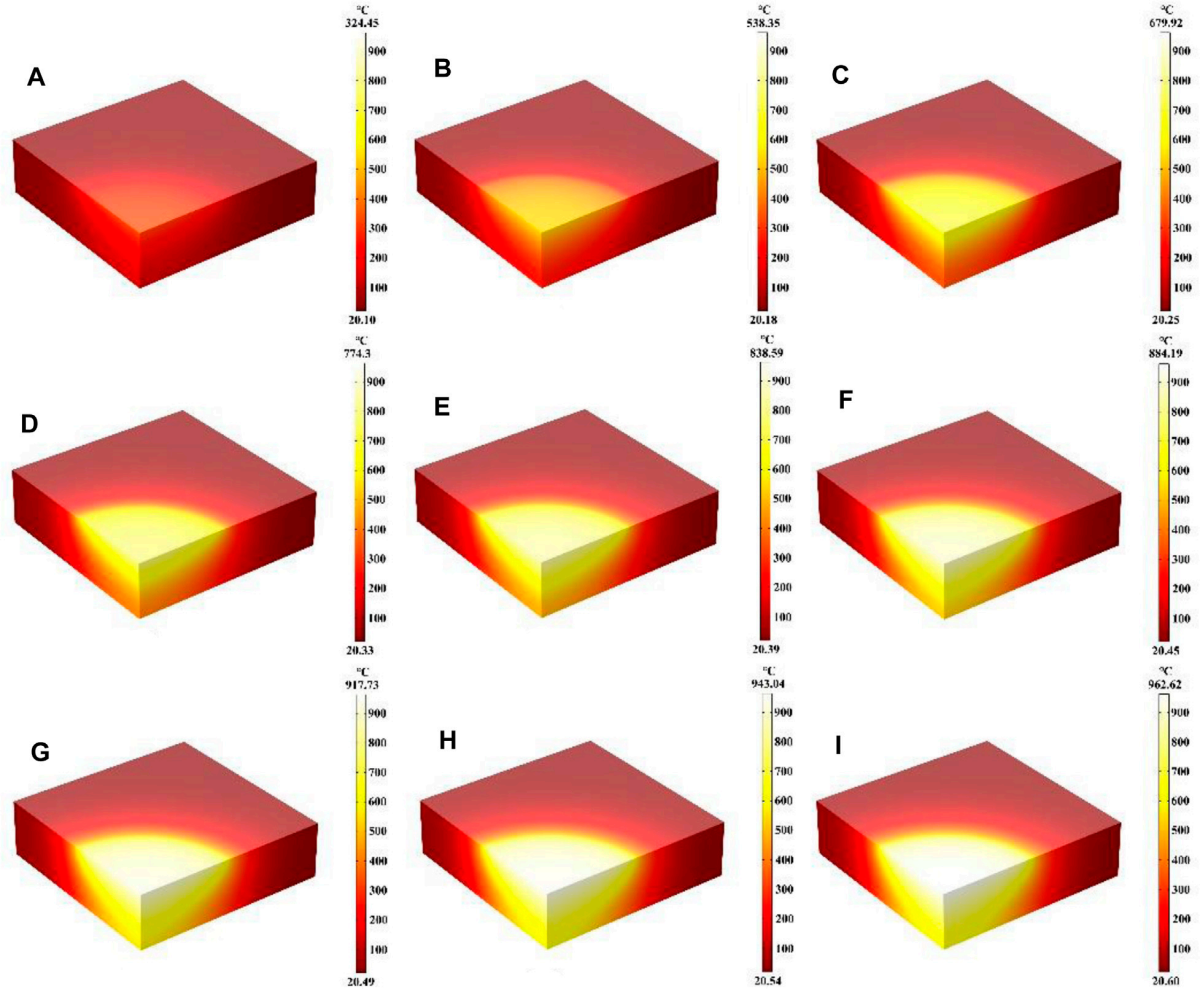
**(A–I)** Are the schematic diagrams of the model temperature distribution under different laser absorption rate, respectively.

In this part, the effect of different laser absorption rates (α) on the heating process were mainly investigated, where the laser heat flux, laser radius and heating time remain unchanged. Figures 4A–I show the temperature field distribution of the quartz material at absorption rates from 0.1 to 0.9 after laser heating for 600 s, respectively. It can be seen that the temperature of the quartz material increases with the increase of the laser absorption rate, and the temperature maximum is located at the center of the quartz material. As the laser absorption rate rises, the temperature gradient distribution within the quartz material also intensifies (Wang D. W. et al., 2022; Li et al., 2024e). When α is over 0.7, the distribution of high temperature regions is transferred to the bottom of the quartz material. Compared with the influence of laser heat flux on the temperature distribution after heating for 600 s the effect of laser absorption on the temperature distribution is less. As the laser absorption rate of the quartz material increases, more laser energy is absorbed by the material at the same laser radius and heat flux, leading to a higher temperature of the quartz material and a greater temperature gradient (Zhu et al., 2022).

**FIGURE 5:**
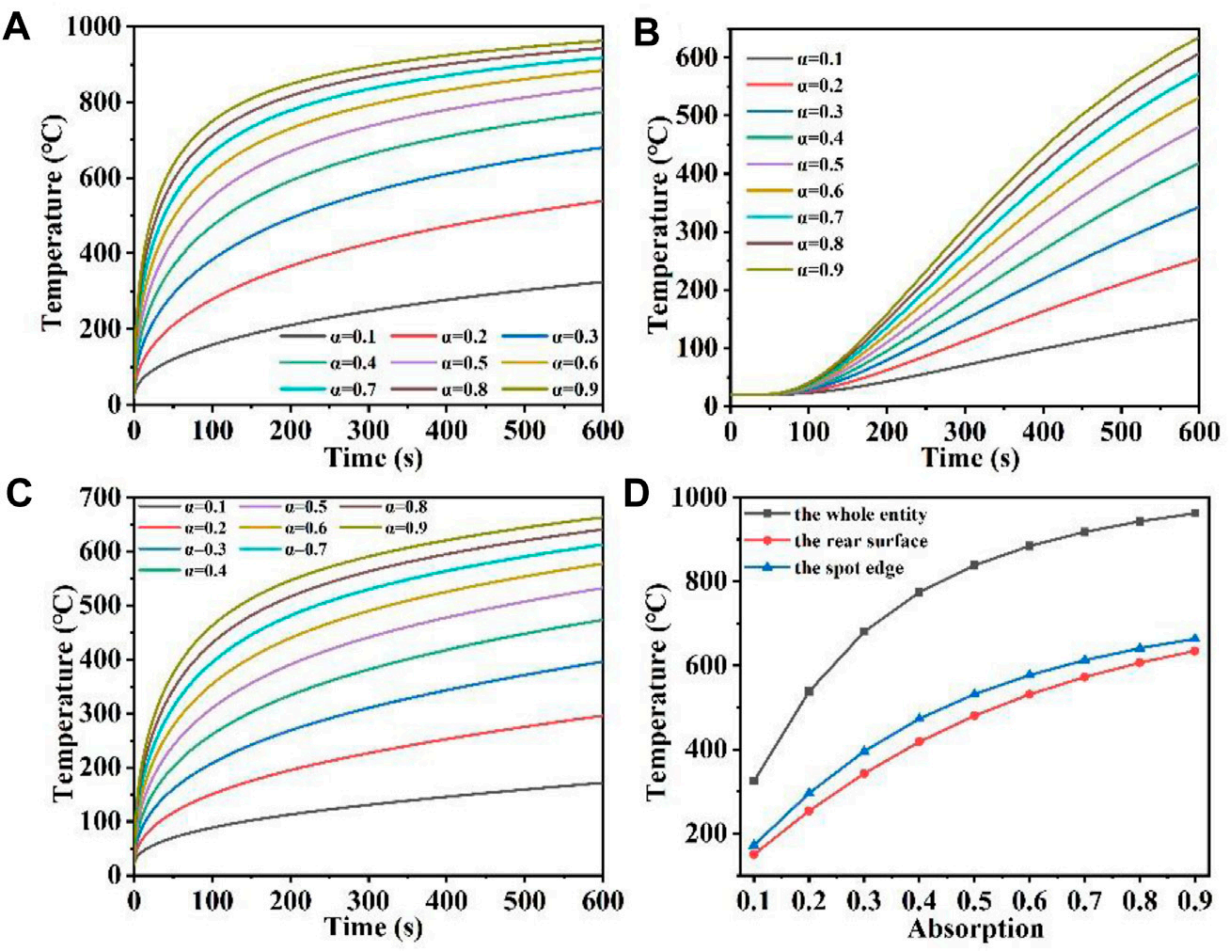
**(A–C)** Are the temperature versus time curves of the whole entity, the rear surface, and the spot edge of the model under different laser absorption, respectively; **(D)** is the temperature versus laser absorption curve under different positions of the model.

We also set the maximum temperature probe of the whole quartz material, the maximum temperature probe of the rear surface and the maximum temperature probe of the spot boundary respectively, and obtained their temperature rise curves over time, as shown in Figures 5A–D. As can be seen from Figure 5A, when the laser absorption rate is large, such as α = 0.9, the temperature first rises rapidly by 900°C, as the surface heat radiation loss intensifies, the temperature rises slowly. When the laser absorption rate is relatively high, for example, α = 0.1, the temperature increases slowly with the increase of irradiation time compared to the higher laser absorption rate, while the temperature increase slows down due to radiation loss. However, the greater the laser absorption rate of the material due to the constant laser spot area and laser heat flux, indicating that the more laser energy incident on the surface of the quartz material, the more laser energy absorbed by the quartz material (Shangguan et al., 2022a; Lei et al., 2024). Figure 5A illustrates that as the laser heat flux and temperature rise rate increase under constant laser irradiation time, the laser absorption rate also increases. A higher incline of the temperature rapidly rising region curve results in a milder slope for the temperature saturation region curve. When α is over 0.7, the overall maximum temperature difference decreases gradually under different laser absorption rates. In addition, Figure 5B shows that when the absorption rate of incident laser is larger, the rear surface maximum temperature is correspondingly higher. Different from the overall maximum temperature in Figure 5A, there is also a hysteresis period for the change of the maximum temperature of the rear surface, and the temperature of the rear surface gradually begins to rise after the irradiation time is over 75 s. In addition, Figure 5C shows that the temperature rise at the edge of the spot under different laser absorption rates is similar to the overall temperature change in Figure 5A. Finally, Figure 5D shows the relationship between the temperature of the whole quartz material, the rear surface and the edge of the spot and the laser absorptivity. The overall temperature distribution is the largest, followed by the temperature at the edge of the spot, and the temperature distribution at the bottom is the smallest (Li et al., 2023b; Jiang et al., 2023). With the increase of laser absorption rate, the temperature increases gradually at different positions. When the laser heat is greater, the higher the temperature of the material after laser heating, because the higher the temperature, the greater the surface heat radiation loss, the temperature rise trend is further slowed down.

### 3.3 The effect of laser radius on laser heating

**FIGURE 6:**
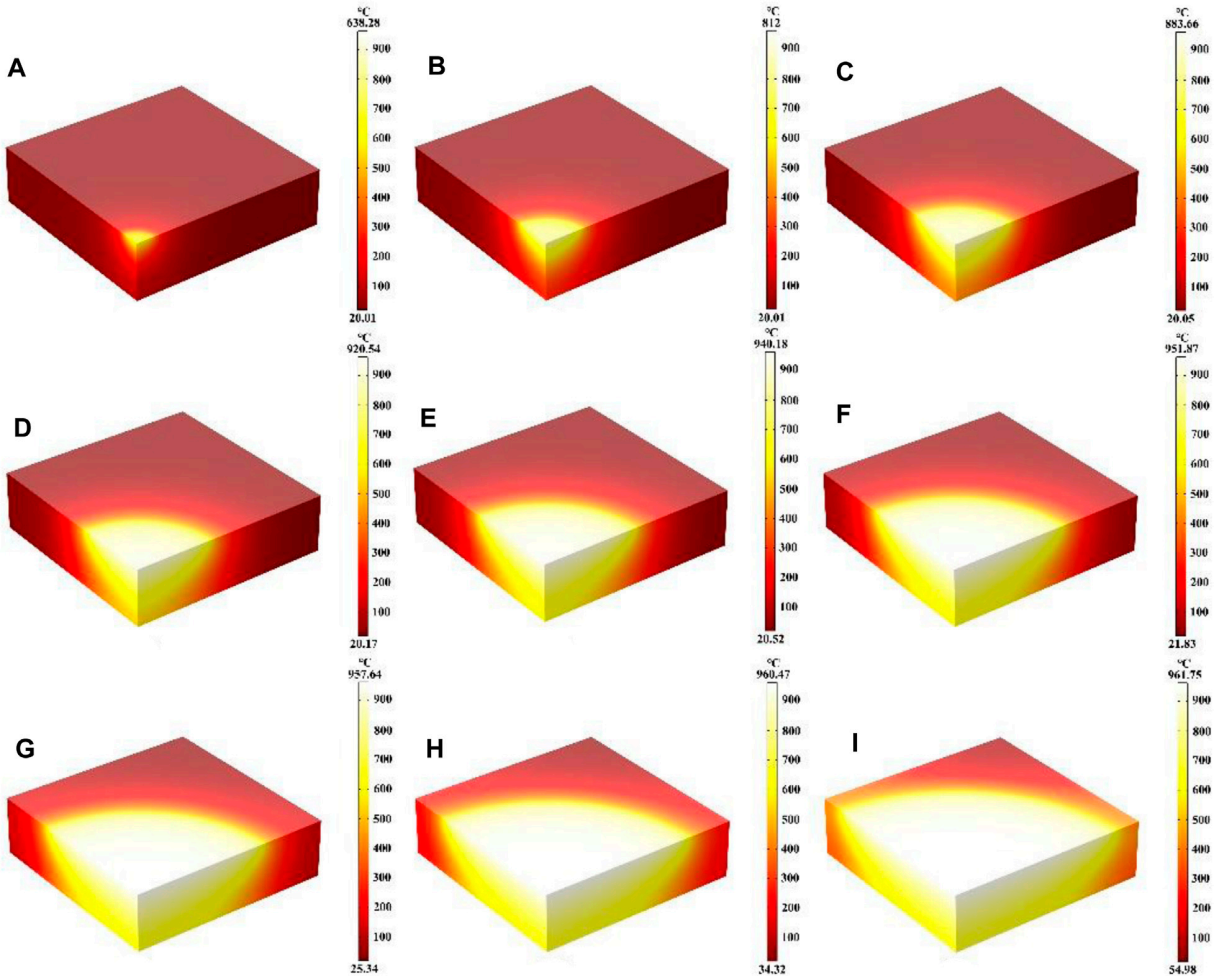
**(A–I)** Are the schematic diagrams of the model temperature distribution under different laser radii.

In this part, the effects of different laser radii on the heating process were mainly investigated, where the laser heat flux, laser absorption rate and heating time of 600 s remain unchanged. Figures 6A–I show the temperature field distribution of the quartz material after laser heating for 600 s at laser radii from 1 cm to 9 cm, respectively. The maximum temperature is located in the center of the quartz material with the increase of the laser radius. The maximum temperature of the quartz material is 638.28°C and 961.75°C when the radius is 1 cm and 9 cm, respectively. When the laser radius changes, according to Eq. 4, the incident laser power also changes, indicating that with the increase of the laser radius, the incident laser power also increases. The more energy is converted into heat energy under the same laser absorption rate. Therefore, the laser radius increases and the overall maximum temperature increases in a macroscopic way (Cvecek et al., 2019).

**FIGURE 7:**
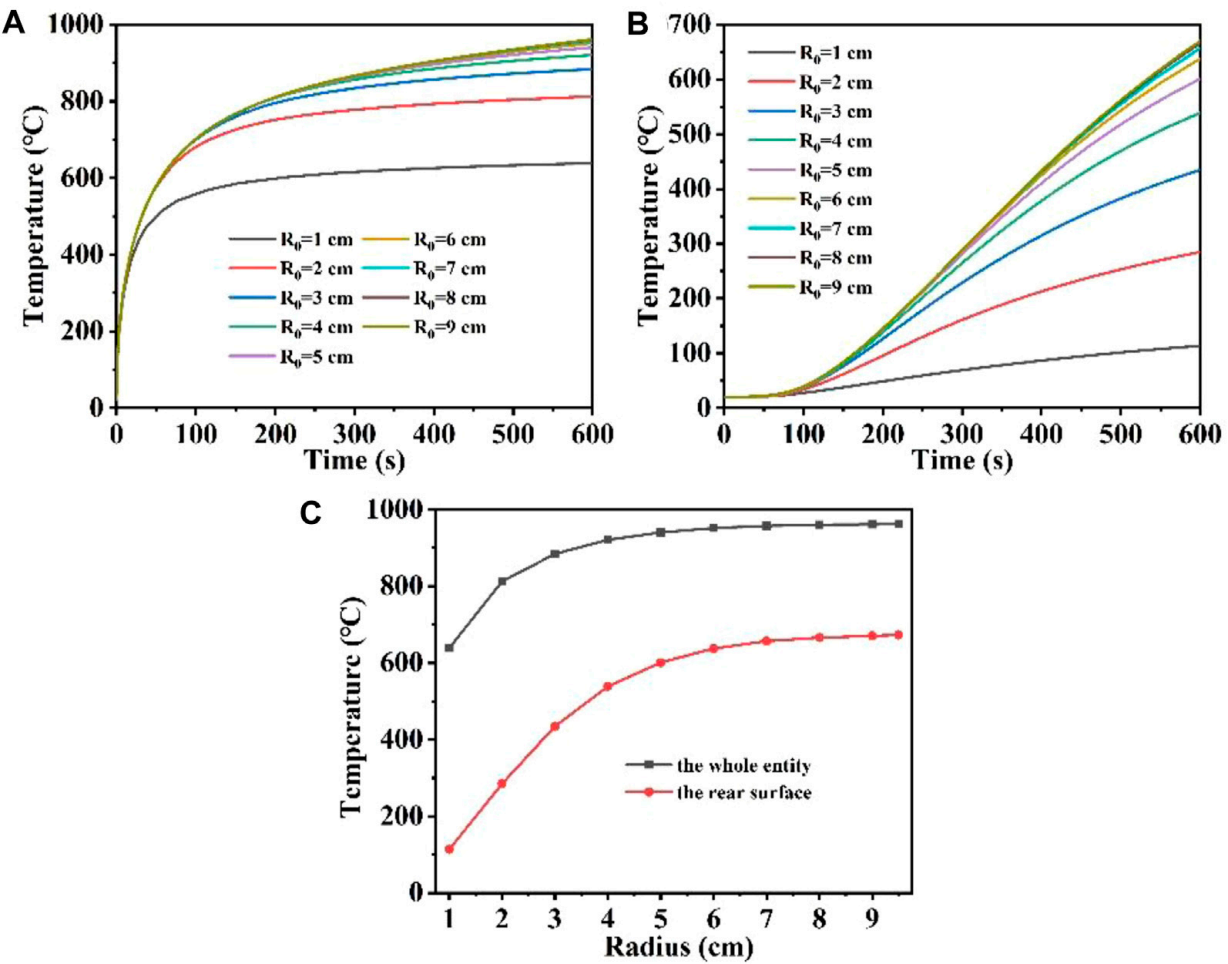
**(A, B)** are the temperature versus time curves of the whole entity, the rear surface of the model under different laser radii, respectively; **(C)** is the temperature versus laser radii curve under different positions of the model.

Figures 7A, B show the maximum temperature versus time variation curves for the whole quartz material and the rear surface at different laser radii, respectively; Figure 7C shows the maximum temperature versus laser radii variation curves for the quartz material at different positions. Figure 7A shows that when the radius is 9 cm, the temperature first rises rapidly by 900°C, as the surface heat radiation loss intensifies, the temperature rises slowly. When the radius of 1 cm, the temperature first rapidly rises by about 600°C compared with a larger laser radius, and the temperature rise is slowed down due to radiation loss. When the laser radius is over 5 cm, the overall maximum temperature difference of the quartz material under different laser radii is very small, and the temperature rise curves almost overlap. In addition, Figure 7B shows that when the radius of the incident laser is larger, the rear surface maximum temperature is correspondingly higher (Shangguan et al., 2022b; Li et al., 2023c). Unlike the overall maximum temperature in Figure 3A, there is also a “hysteresis period” for the change of the rear surface maximum temperature, and the temperature at the bottom gradually begins to rise when the irradiation time is over 100 s. When the laser radius is over 6 cm, the overall maximum temperature difference of the quartz material under different laser radii is very small, and the temperature rise curves almost overlap (Wu et al., 2024). In order to analyze the curves overlap more directly, Figure 7C was obtained to show the relationship between the maximum temperature of the whole quartz material, the rear surface and the laser radius. As the laser radius increases, both the overall maximum temperature and the maximum temperature rise on the rear surface first increase and then stabilize at a constant value (Lin et al., 2023). This is because with the increase of the laser radius, the laser power also increases under the condition of constant laser heat flux, according to Eq. 4 (Shangguan et al., 2022c; Sun et al., 2022; Lei et al., 2024). However, the larger the radius, the larger the laser area, the greater the distribution of high temperature regions, the greater the heat radiation loss, further slowing down the temperature rise, so whether it is the maximum temperature of the whole quartz material, the rear surface, increase initially and then stabilize at a constant value as the laser radius increases.

### 3.4 The effect of quartz material thickness on laser heating

**FIGURE 8:**
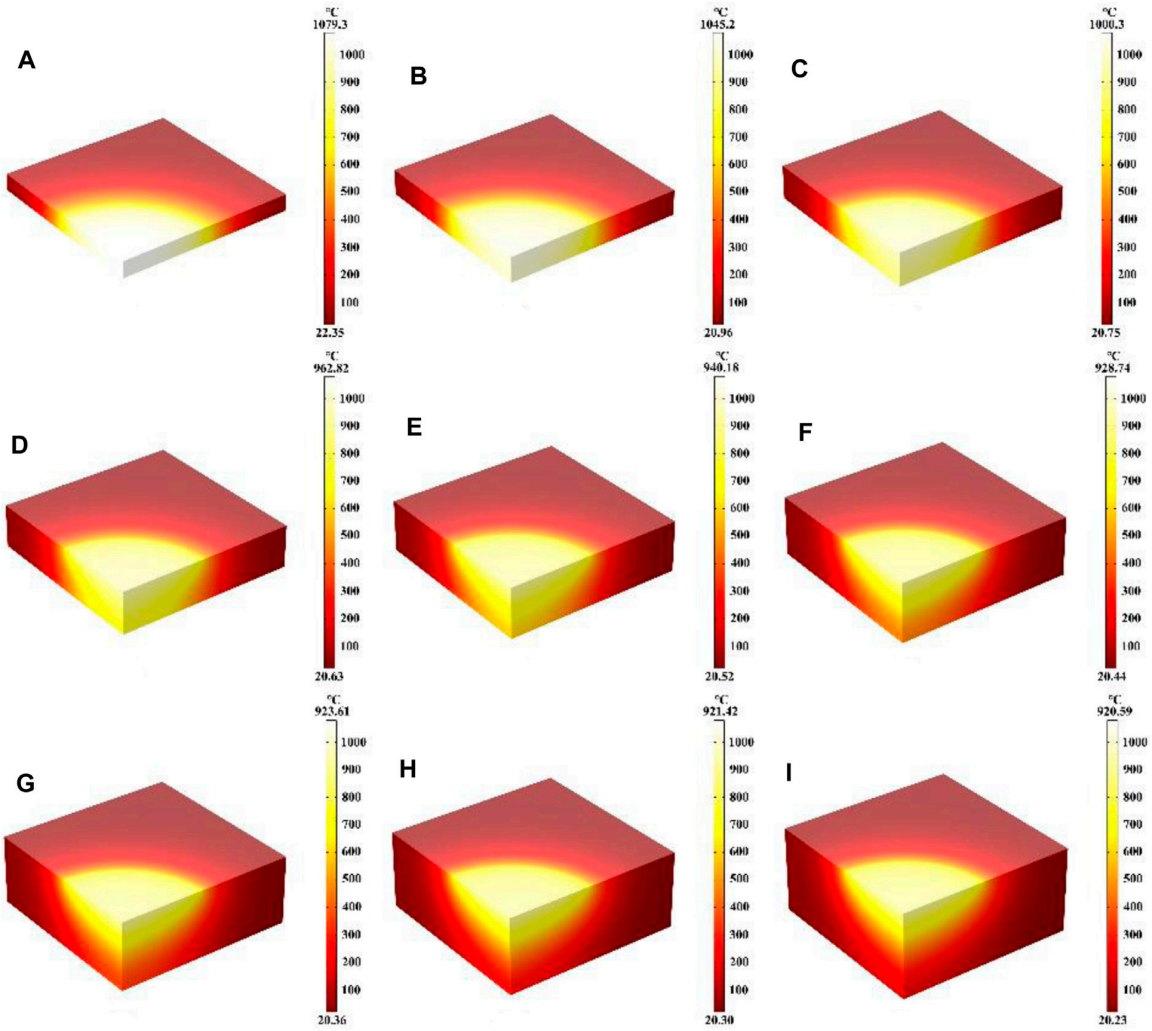
**(A–I)** Are the schematic diagrams of the model temperature distribution under different thicknesses, respectively.

In this part, the effect of different quartz material thicknesses on the heating process was mainly investigated, where the laser heat flux, laser absorption rate, laser radius and heating time of 600 s remain unchanged. Figures 8A–I show the temperature field distribution of the quartz material after laser heating for 600 s at material thicknesses from 1 cm to 5 cm, respectively. As the thickness of the quartz material increases, the maximum temperature of the material decreases with the highest temperature being at the center of the quartz material. When the thicknesses are 1 cm and 5 cm, the maximum temperature of the quartz material are 1,079.3°C and 920.59°C. Thinner quartz material results in a more pronounced and higher temperature range, whereas thicker quartz material leads to a less noticeable temperature range compared to thinner variants. This occurs primarily due to the fact that when the quartz material is thinner, the absorbed laser heat builds up rapidly within the material, leading to an overall higher temperature of the quartz (Li et al., 2023d; Lei et al., 2024).

**FIGURE 9:**
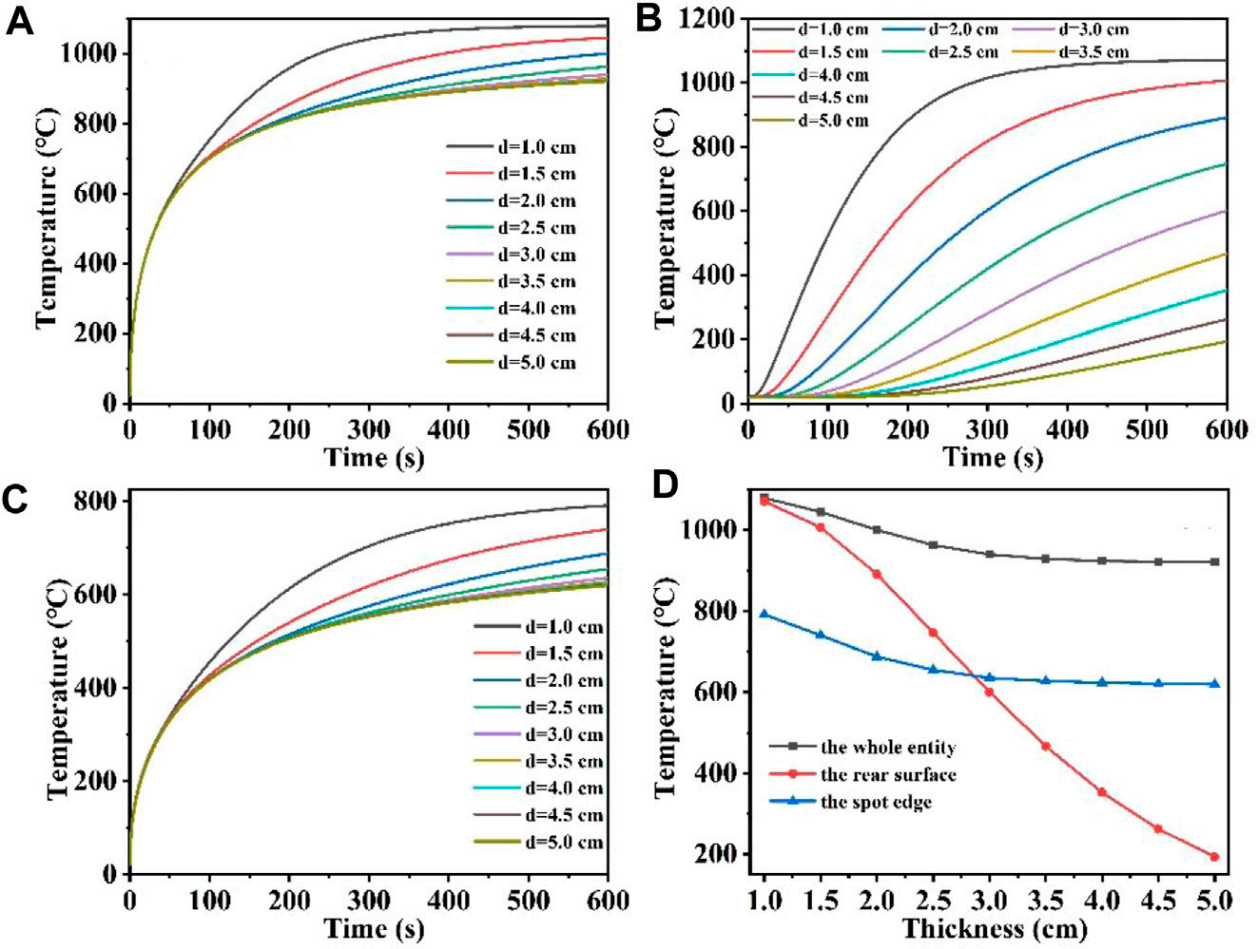
**(A–C)** Are the temperature versus time curves of the whole entity, the rear surface, and the spot edge of the model under different thicknesses, respectively; **(D)** is the temperature versus thickness curve under different positions of the model.

Figures 9A–C shows the maximum temperature versus time at different quartz material thicknesses for the whole quartz material, the rear surface and the edge of the spot, respectively; Figure 9D shows the maximum temperature versus quartz material thicknesses at different positions. Figure 9 shows that when the thickness of the quartz material is 5 cm, the temperature first rises rapidly by 500°C, as the surface heat radiation loss intensifies, the temperature rises slowly. When the quartz material is thin, for example, at a thickness of 1 cm, the temperature first rises rapidly by 1,000°C. When the quartz material thickness is over 3 cm, the overall maximum temperature difference between different thicknesses is minimal, and the temperature rise curves nearly coincide. In addition, Figure 9B shows that when the quartz material is thinner, the maximum temperature at the bottom is correspondingly higher. Unlike other conditions, the thicker the quartz material, the maximum temperature change at the bottom will appear “hysteresis period,” and the “hysteresis period” will be longer with the increase of quartz material thickness (Benyounis et al., 2005; Zheng et al., 2022). Figure 9C shows a similar temperature change as Figure 9A. And the temperature at the edge of the spot is often lower than the temperature inside the laser spot under the same laser heat flux. In order to analyze the curve overlap more directly, Figure 9D is obtained to show the relationship between the maximum temperature of the whole quartz material, the rear surface and the edge of the spot and the quartz material thickness. As the thickness of the quartz material increases, the maximum temperature at both the center and the edge of the spot initially decreases and eventually stabilizes at a constant value. This occurs primarily because thinner quartz material allows absorbed laser heat to accumulate rapidly within it, leading to an overall increase in temperature (Yu et al., 2022; Zhu et al., 2023). Conversely, thicker quartz material absorbs the same amount of laser heat as thinner material but distributes it over a larger surface area. In addition, the radiation loss of heat to the air is fixed, resulting in the maximum temperature of the whole and the edge of the spot gradually tending to a constant value with increasing thickness (Wang et al., 2022b; Zhang C. et al., 2022). In addition, the maximum temperature at the bottom gradually decreases with increasing thickness, because under the same laser conditions, the thicker the quartz material, the longer the heat transfer process takes, coupled with convection and radiation heat loss, the heat reaching the bottom of the quartz material is less than that of the thinner quartz material (Ma et al., 2023b; Lv et al., 2024). Therefore, the maximum bottom temperature decreases with the increase of quartz material thickness.

### 3.5 The effect of laser irradiation time on laser heating

**FIGURE 10:**
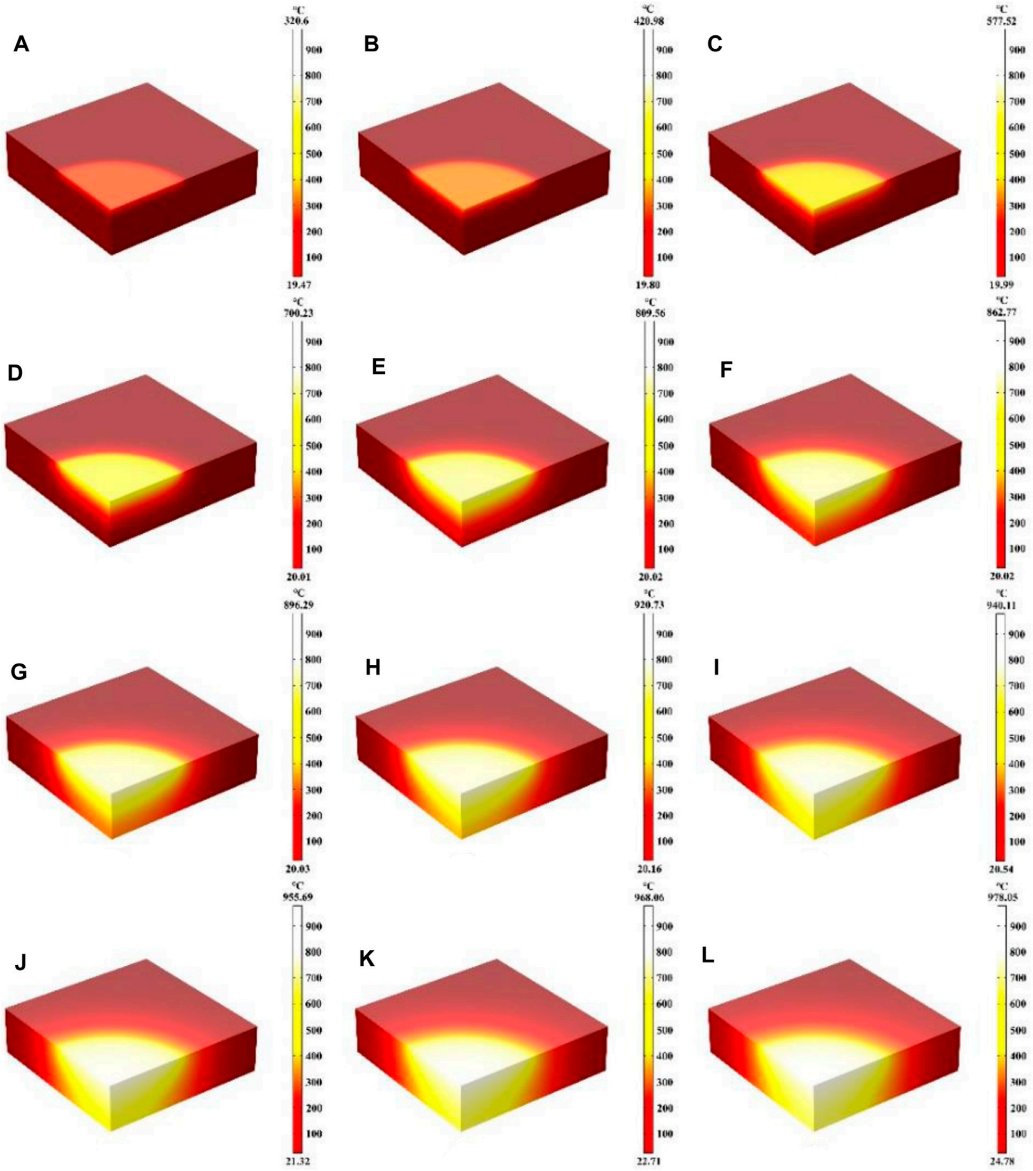
**(A–L)** Are the schematic diagrams of the model temperature distribution under different laser irradiation times (5 s, 10 s, 20 s, 100 s, 200 s, 300 s, 400 s, 500 s, 600 s, 700 s, 800 s, 900 s, respectively).

**FIGURE 11:**
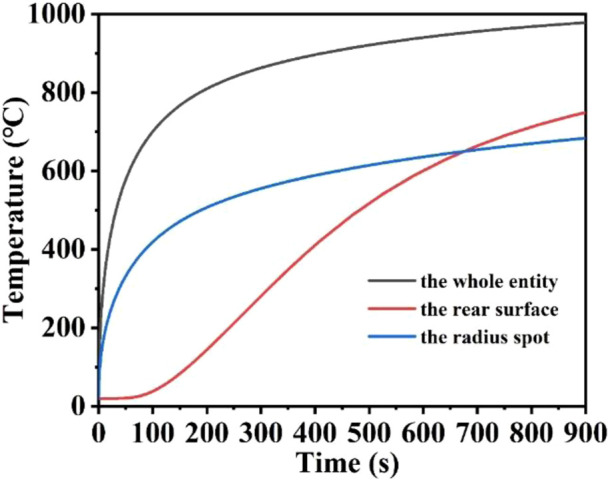
The temperature versus laser irradiation time curves of the whole entity, the rear surface, and the spot edge of the model.

In this part, the effect of laser heating time on the heating process is mainly investigated, where the laser heat flux, laser absorption rate and laser radius remain unchanged. Figures 10A–I represent the schematic diagrams of the temperature field distribution of the quartz material after laser heating from 5 s to 900 s, respectively. When the heating time is 5 s, the quartz material is rapidly heated to 320.6°C, and when the heating time is 200 s, the quartz material is heated to 899.56°C, and the higher temperature distribution region is further expanded with the increase of heating time. When the heating time is 600 s, an expansion of the higher temperature distribution area reaching the bottom of the quartz material, and the maximum temperature is 940.11°C, and when the heating time is 900 s, the quartz material is heated to 978.05°C. At the beginning of laser heating, the maximum temperature of the quartz material rises rapidly, and with the increase of heating time, the thermal radiation loss becomes larger and larger, further slowing down the temperature rise (Jiang et al., 2021; Wang et al., 2022c). Therefore, the maximum temperature of the quartz material increases slowly during the heating time of 600–900 s. Figure 11 shows the maximum temperature of the whole quartz material, the rear surface and the edge of the spot as a result of changing over time. Within 200 s before heating, the overall temperature and the edge of the spot rise rapidly. With the increase of temperature, the heat radiation loss is greater, and the temperature rise is further slowed down, and the temperature rise curve is gradually flattened (Zheng et al., 2024a; Lu et al., 2024). Due to the stepped distribution of the flat-top laser at the edge of the spot, heat conduction is easy to occur at the edge of the spot, and the temperature at the edge of the spot is often lower than the temperature inside the laser spot under the same laser heat flux. In addition, there is a “hysteresis period” on the rear surface of the quartz material (Hu et al., 2020; Yan et al., 2023; Zheng et al., 2024b). When the irradiation time is over 60 s, the bottom temperature gradually begins to rise. When the heating time is over 700 s, the highest temperature of the lower surface is over the maximum temperature of the edge of the spot, and the energy absorbed by the laser above is absorbed and converted into heat. As the irradiation time prolongs, heat conduction gradually shifts the energy to the underside of the quartz material.

## 4 Conclusion

A 3D quarter-symmetric laser heating quartz material geometry model by using nonlinear transient finite element method was established, and its transient temperature field distribution of the quartz material after being heated by a 1,064 nm continuous laser was investigated. In addition, the influence of different laser parameters (laser spot radius, heat flux and irradiation time), material parameters (material thickness, material absorption rate of laser) on the thermal effect of heating quartz material were also studied. When the laser heat flux is 20 W/cm^2^, the diameter of the laser spot is 10 cm, the irradiation time is 600 s and the thickness is 4 cm, the temperature after laser heating can reach 940.18°C, which is far lower than the melting point, reflecting the excellent thermal insulation performance of the material. The higher the laser heat flux, the higher the absorption rate, the larger the laser radius and the smaller the thickness of the quartz material, the longer the laser irradiation time, the higher the temperature the quartz material can reach. In addition, the temperature maximum probes were set at the overall model, spot edge and rear surface respectively, and their temperature rise curves with time were obtained. It is also found that there is a significant hysteresis period for the rear surface temperature change of the quartz material compared with the overall temperature change due to heat conduction. Finally, the research method proposed in this paper can not only be used for the laser heating research of other non-transparent materials, but also guide the actual laser heating process according to the research results, saving experimental resources.

## Data Availability

The original contributions presented in the study are included in the article/Supplementary Material, further inquiries can be directed to the corresponding author.
